# Simultaneous and Rapid Determination of Six Tyrosine Kinase Inhibitors in Patients with Non-Small Cell Lung Cancer Using HPLC-MS/MS

**DOI:** 10.1155/2021/5524361

**Published:** 2021-09-17

**Authors:** Yanping Liu, Hua Liu, Zhewei Xia, Zhipeng Wang, Yunlei Yun, Guanying Zhang, Lifeng Huang, Shouhong Gao, Wansheng Chen

**Affiliations:** ^1^Department of Pharmacy, Changzheng Hospital, Second Military Medical University, Shanghai 200003, China; ^2^Department of Pharmacy, The Affliated Suzhou Science and Technology Town Hospital of Nanjing Medical University, Suzhou 215153, China; ^3^Department of Pharmacy, Taixing People's Hospital, Taizhou, Jiangsu 225400, China; ^4^Pharmaceutical Analysis and Testing Center, School of Pharmacy, The Second Military Medical University, Shanghai 200003, China

## Abstract

**Objective:**

To develop a new method for quantitatively analyzing six tyrosine kinase inhibitors (gefitinib, erlotinib, icotinib, afatinib, osimertinib, and crizotinib) used in the treatment of non-small cell lung cancer (NSCLC) by liquid chromatography-tandem mass spectrometry (LC-MS/MS).

**Methods:**

The analytes were detected in the selected reaction monitoring mode on a triple quadrupole mass spectrometer with the positive ionization mode. Carbamazepine was utilized as the internal standard. The pretreatment of the plasma sample was completed based on protein precipitation with acetonitrile, and the analytes were separated on an Agilent Zorbax SB-C18 reversed-phase column (2.1 mm × 100 mm, 3.5 *μ*m, Agilent, USA) using gradient elution. The mobile phase consisted of 0.1% formic acid in water (phase A) and 0.1% formic acid in acetonitrile (phase B). The flow rate was 0.3 mL/min, and the injection volume was 5 *μ*L. The column temperature was set and maintained at 35°C.

**Results:**

The calibration curves were linear over the range from 5.0 to 1000.0 ng/mL for gefitinib, crizotinib, and osimertinib; from 50.0 to 4000.0 ng/mL for icotinib and erlotinib; and from 5.0 to 400.0 ng/mL for afatinib. Linear correlation coefficients were >0.990 for all regression curves. The intra- and interday accuracy and precision of the method were within ±15.0% and not more than 15.0%, respectively. The mean recovery of all the analytes ranged from 70.18% to 110.76%, the matrix effect was from 88.85% to 127.58%, and stability was within ±15.0%.

**Conclusion:**

This newly developed method was sensitive, simple, and robust and could be used in therapeutic drug monitoring of six tyrosine kinase inhibitors in NSCLC patients.

## 1. Introduction

Lung cancer is one of the most common malignant tumors in the world, among which the number of patients with non-small cell lung cancer (NSCLC) is about 85–90%, and 14% of cancer deaths in the world are caused by NSCLC [1]. In the last 50 years, treatments for NSCLC mainly included chemical drugs. Chemotherapy regimens of cisplatin, vincristine combined with cisplatin, and paclitaxel have prolonged the survival time and improved the quality of life of lung cancer patients, but the 5-year overall survival rate is about 5% [2]. In recent years, tumor treatment has developed rapidly, and oral small-molecule targeting drugs have emerged successively. These oral small-molecule targeting drugs bind to certain receptors on the tumor cells, thereby inhibiting downstream tyrosine kinase signaling and tumor proliferation. The primary small-molecule target drugs mainly target epidermal growth factor receptor (EGFR) and anaplastic lymphoma kinase (ALK). EGFR and ALK mutations occur mainly in nonsmoking NSCLC patients, and nonsmoking patients also have higher survival rates than smokers [3]. In newly treated patients with EGFR-mutated NSCLC, compared with platinum chemotherapy, first-generation EGFR inhibitors (gefitinib and erlotinib) or second-generation EGFR inhibitors (afatinib and icotinib) had significant higher survival and better quality of life [4]. However, EGFR (T790M) secondary mutations occur in approximately 50% of patients with progressive lung cancer after 9–13 months of treatment. Osimertinib, the third-generation EGFR inhibitor, showed a greater survival advantage in patients with secondary mutations at EGFR (T790M) than platinum-based chemotherapy [5]. Crizotinib significantly improved the quality of life in patients of nonprogressive lung cancer or NSCLC of ALK rearrangement [6].

Molecular targeted drugs are all given orally on fixed dose, which have good efficacy, but there are also many adverse reactions. Adverse reactions are one of the factors leading to interruption of treatment. Many pharmacokinetic studies have reported the relationship between clinical efficacy or adverse reactions and plasma exposure [7–10]. However, there were significant individual differences in plasma exposure levels. The therapeutic window of these drugs was narrow, and there were significant differences in pharmacokinetics among individual patients [11]; therefore, these drugs are candidates for therapeutic drug monitoring (TDM). TDM is a dose optimization strategy to achieve faster and more effective clinical efficacy. The use of the rapid and sensitive LC-MS/MS detection method to determine the concentration of drugs in human blood or other body fluids can effectively adjust the dosage of drugs, improve the therapeutic effect of drugs, reduce the adverse reactions, and ensure the rational use of drugs. In order to solve this problem, several LC-MS/MS methods have been developed [12–15], but these methods have high requirements for instruments, complicated operation, and long analysis time, which hinder the clinical application. Therefore, the purpose of this study was to establish a simple, rapid, and sensitive method for the simultaneous determination of six TKIs in human plasma and verify its clinical application.

## 2. Materials and Methods

### 2.1. Chemicals and Reagents

The analytes including gefitinib (lot: F1102AS), icotinib (lot: J0615A), afatinib (lot: M0320A), erlotinib (lot: J0615A), crizotinib (lot: A0320A), and carbamazepine (internal standard, IS) (lot: M1001AS) were supplied by Meilun Biotech Co., Ltd. (Dalian City, China). Osimertinib (lot: 1-NJL-79-1) was purchased from Toronto Research Chemicals (Toronto, Canada). Mass spectrometric reagents methanol and acetonitrile were obtained from Merck (Merck Company, Darmstadt, Germany).

Formic acid was purchased from Tedia Company Inc. (Tedia, Fairfield, OH, USA). Isopropanol reagent was purchased from Shanghai Titan Technology Co., Ltd. (Titan, Shanghai, China). Distilled water was purchased from Watsons Distilled Water Co., Ltd. (Watsons, Guangzhou, China). Human blank plasma was donated by healthy volunteers in our laboratory (Shanghai, China).

### 2.2. Mass Spectrometry

The experiment was performed on an Agilent 1200 series HPLC system, consisting of an online degasser, a binary pump, an autosampler, and a column oven and interfaced to an Agilent 6410A triple quadrupole mass spectrometer equipped, in which the ionization source is the electrospray ionization source (ESI source, Agilent Technologies, USA). The data were processed using Agilent MassHunter data processing software (version B.01.04; Agilent Technologies, USA).

### 2.3. Liquid Chromatographic Conditions

All analytes were performed on a Zorbax SB-C18 analytical column (2.1 mm × 100 mm, 3.5 *μ*m, Agilent, USA). The mobile phases contained 0.1% formic acid in water (phase A) and 0.1% formic acid in acetonitrile (phase B), and the flow rate was set at 0.3 mL/min. The gradient program started at 30% B, increased gradually to 90% B in 3 min, and then was held at 90% B until 7 min. The pastime was 5 min. The column temperature was set at 35°C, and the autosampler was maintained at room temperature. The volume injected into the chromatographic system was 5 *μ*L.

### 2.4. Mass Spectrometry Conditions

**Figure 1:**
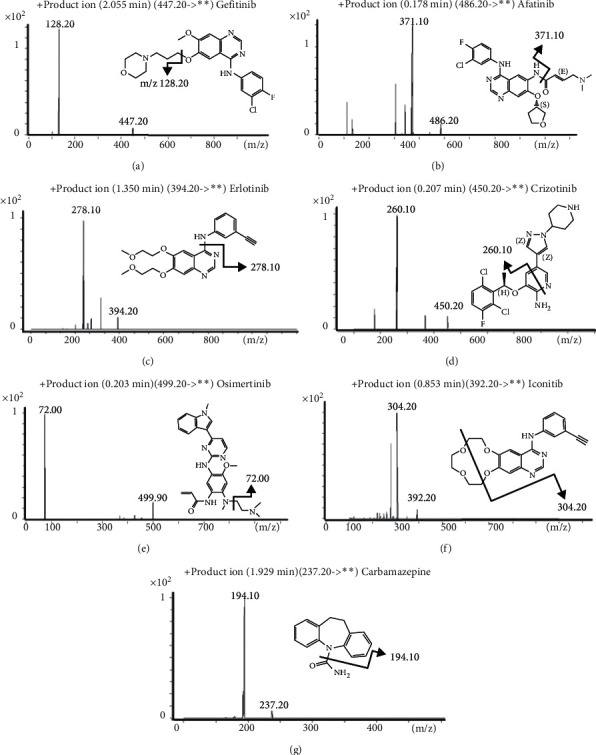
Product ion chromatograms and fragment structures of six TKIs. (a) Gefitinib. (b) Afatinib. (c) Erlotinib. (d) Crizotinib. (e) Osimertinib. (f) Icotinib. (g) Carbamazepine (IS).

All analytes were collected under the multiple reaction monitoring (MRM) and positive ionization mode (Figure 1). The mass spectrum parameters were as follows: HPLC flow rate of 250 L/min, sheath gas flow rate of 12 L/min, and temperature of 250°C. Nozzle voltage is 500 V. The atomizer pressure is 45 psi. Capillary voltage is 4000 V. The drying gas and atomizing gas are nitrogen, the flow rate of drying gas is 5 L/min, and the temperature is 350°C.  Table 1 shows the optimized MRM parameters for six analytes and IS.

### 2.5. Preparation of Standard and Quality Control Samples

The stock solutions of all analytes were prepared, respectively, in 70% methanol (methanol-water, 70 : 30, V/V), and 2.05, 2.02, 1.99, 2.02, 2.08, and 2.00 mg of gefitinib, icotinib, afatinib, erlotinib, crizotinib, and osimertinib were accurately weighed and dissolved to obtain 1.0 mg/mL for all of them. The stock solutions were aliquoted and stored at −80°C. The stock solution of analytes was further diluted with 10% methanol (methanol-water, 10 : 90, V/V) to obtain combined work solutions at the following concentrations: 100, 200, 500, 1000, 2000, 5000, and 10000 ng/mL for gefitinib, crizotinib, and osimertinib; 500, 1000, 2000, 5000, 10000, 20000, and 40000 ng/mL for icotinib and erlotinib; and 50, 100, 200, 500, 1000, 2000, and 4000 ng/mL for afatinib; they were diluted with blank human plasma 10 times, and their concentrations ranged from 10 to 1000 ng/mL for gefitinib, crizotinib, and osimertinib; from 50 to 4000 ng/mL for icotinib and erlotinib; and from 5 to 400 ng/mL for afatinib. Quality control (QC) samples were also prepared in the same way for each TK, and their concentrations were set at 20.0, 100.0, and 500.0 ng/mL for gefitinib, crizotinib, and osimertinib; at 100.0, 500.0, and 2000.0 ng/mL for icotinib and erlotinib; and at 10.0, 50.0, and 200.0 ng/mL for afatinib. The stock solutions were stored at −80°C. They were brought to room temperature (25°C) for thaw before pretreatment. For IS stock solution, 1.99 mg carbamazepine was dissolved in 70%methanol and stored at −80°C after aliquot. The IS work solution was freshly prepared with acetonitrile at a concentration of 100 ng/mL for carbamazepine and stored at −20°C.

### 2.6. Sample Pretreatment

The samples were prepared as follows: for all analytes, sample pretreatment was performed by protein precipitation. The blood sample (50 *μ*L) was transferred to a 1.5 mL Eppendorf tube prior to spiking with 100 *μ*L of acetonitrile (containing 100 ng/ml of IS solution). After being vortexed for 1 min, the mixture was centrifuged at 14500 ×g for 15 mins at room temperature. Then, 100 *μ*L of the supernatant solution was transferred to a 1.5 mL Eppendorf tube prior to adding 200 *μ*L mobile phase (A : B, 70 : 30, V/V). The sample was centrifuged at 14500 rpm for another 15 mins after being vortexed for 1 min at room temperature. Then, 5 *μ*L of the supernatant solution was directly injected into the HPLC-MS/MS system for analysis.

### 2.7. Human Sample

This research was approved by and performed at Changzheng Hospital (Shanghai, China) from March 2019 to May 2019. Blood samples were collected in EDTA-3K tubes from NSCLC patients after treatment with TKIs, and a total of 25 patients with NSCLC were enrolled in this study. And 3 mL venous blood samples were collected after a food fasting overnight, gently mixed after the TKI in NSCLC patients had reached steady concentration (two weeks after the first dose), and then centrifuged at 3000 ×g for 10 minutes. The plasma was harvested and measured by the above method.

### 2.8. Method Validation

According to the Chinese Pharmacopoeia (version 2010) and FDA guidelines [16], method validation includes specificity, lower limit of quantitation (LLOQ), linearity, inter- and intraprecision and accuracy, carryover, extraction recovery, matrix effect, and stability.

## 3. Results and Discussion

### 3.1. Chromatography Condition Optimization

In this experiment, some universal columns containing Agilent Zorbax SB-C18 (2.1 mm × 100 mm, 3.5 *μ*m), Waters XSELECT™ HSS PFP (2.1 mm × 100 mm, 3.5 *μ*m), and XBridge™ BEH C18 (2.1 mm × 50 mm, 2.5 *μ*m) were tested for their retention and separation ability. By comparing their chromatographic features (such as resolution, retention time, response value, and peak shape), the results showed a better peak shape and response on the Agilent Zorbax SB-C18 (2.1 mm × 100 mm, 3.5 *μ*m) column and achieved complete separation of analytes in a short time from endogenous interferents. Therefore, the Agilent Zorbax SB-C18 (2.1 mm × 100 mm, 3.5 *μ*m) column was selected for the development of the method.

Different mobile phase additives (such as formic acid and ammonium acetate) were added to improve the peak shape, response, and retention time. When ammonium acetate was added to the mobile phase, the responses of all analytes were low, and the peak shape was poor. And then, retention time and separation effect were gained after testing with different ratios of formic acid (FA) (0.05%, 0.1%, and 0.2% FA) in the mobile phase. It was found that when 0.1% FA was added to the water phase, the response of analytes could be significantly increased.

### 3.2. Sample Pretreatment Optimization

Protein precipitation, solid-phase extraction (SPE), and liquid-liquid extraction are the main methods used in laboratory sample pretreatment. The protein precipitation method is a simple, economical, and time-saving method, while the cost of liquid-liquid extraction and solid-phase extraction is relatively higher. Therefore, the protein precipitation method is first tested. Acetonitrile and methanol were used as precipitating agents to remove protein from plasma. By comparing the extraction recovery and matrix effect of acetonitrile and methanol in different proportions, it was found that the ratio of acetonitrile to the sample is 2 : 1, and the extraction recovery is higher. In order to further purify the sample and reduce impurities in the sample, the supernatant after centrifugation was further treated. An equal volume of aqueous phase, organic phase, and their mixtures in different ratios was added to the supernatant. Results showed that the ratio of the mixed mobile phase is 7 : 3 (V : V), and the extraction recovery and peak shape are relatively ideal. In the end, comparing the extraction recovery and matrix effect of the supernatant and mixed mobile phase in different proportions, results found that the ratio of the mixed mobile phase to the supernatant is 2 : 1 (V : V), and the extraction recovery is higher. The pretreatment method is simple and economical, which can basically meet the requirements of this experiment and has good practicability.

### 3.3. Method Validation

#### 3.3.1. Specificity

**Figure 2:**
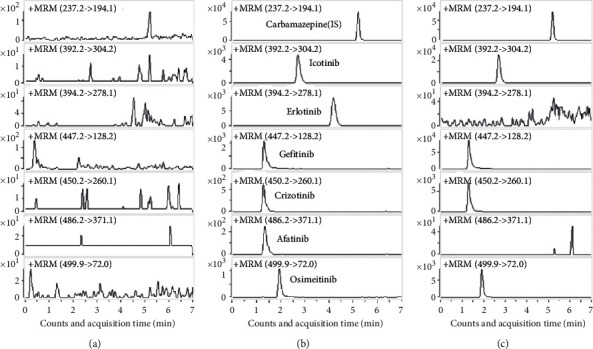
Representative MRM chromatograms of six TKIs: (a) blank sample; (b) blank sample spiked with the LLOQ concentration of six TKIs and IS; (c) real sample concentration (icotinib: 504 ng/mL, erlotinib: ND, gefitinib: 623 ng/mL, crizotinib: 327 ng/mL, afatinib: ND, osimertinib: 250 ng/mL, and IS: 100 ng/mL). ND = not detected.

Comparisons of specificity from blank, IS, and six analytes spiked and clinical real samples (Figure 2) indicated no significant interferences at the same retentiontimes of the analytes and IS. The retention time of six TKIs and IS is as follows: IS: 5.2 min, iconitib: 2.7 min, erlotinib: 4.2 min, gefitinib: 1.3 min, crizotinib: 1.3 min, afatinib: 1.3 min, and osimertinib: 1.9 min.

#### 3.3.2. Linearity

Through the construction of calibration curves, the linear correlation coefficients (*R*) of all analytes were greater than 0.990 under the weighing coefficient of 1/*χ*^2^. The linear correlation coefficients (*R*) were more than 0.990 for all analytes. Results of regression equations for the calibration curves are presented in Table 2. The LLOQ was 10 ng/mL in the human plasma matrix of gefitinib, crizotinib, and osimertinib, 50 ng/mL in iconitib and erlotinib, and 5 ng/mL in afatinib, which were also in accordance with the accuracy within ±20% and precision less than 20%. The LLOQ and accuracy assessment results are summarized in Table 3.

#### 3.3.3. Matrix Effect and Extraction Recovery

The matrix effect and extraction recovery of samples (low, middle, and high) were investigated. The results showed that the matrix effect of the analytes was between 96.83% and 114.09%, and the recovery was between 76.66% and 97.18%. The RSD (%) of the matrix effect and extraction recovery factors was less than 15%. The results are shown in Table 4.

#### 3.3.4. Precision and Accuracy

Three levels of QC samples (low, middle, and high) were chosen to analyze the inter- and intra-accuracy and precision. The results of accuracy with inter- and intra-accuracy were 85.36% to 111.38% and 85.24% to 113.04%, and the inter- and intraprecision were 1.02% to 5.41% and 0.33% to 4.97%.  Table 5 summarizes the inter- and intraday accuracy and precision for the analytes.

#### 3.3.5. Stability

Short-term stability (25°C in room temperature for 12 h and in the autosampler for 24 h), long-term stability (1 month at −80°C), and three freeze-thaw cycles' stability were determined for all analytes in triplicate at each of the low, middle, and high concentrations. Results of stability are shown in Table 6.

### 3.4. Application of Clinical Samples Treated by TKIs

**Figure 3:**
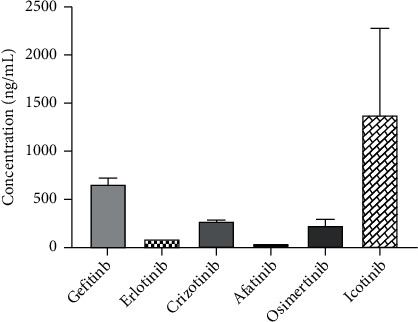
Drug concentration distribution of NSCLC patients with 25 cases.

To test the applicability of this method, 25 plasma samples were collected from 25 NSCLC patients who were treated with one of six drugs. Gefitinib (250 mg qd), erlotinib (150 mg qd), icotinib (125 mg tid), afatinib (40 mg qd), osimertinib (80 mg qd), and crizotinib (250 mg bid) at standard doses at Changzheng Hospital were prescribed according to clinical diagnosis. Nine (erlotinib), seven (osimertinib), three (gefitinib), three (crizotinib), two (afatinib), and one (erlotinib) plasma samples were collected from patients, and the time points were designed in 7–14 days after administration (trough concentration). The TKIs were quantitatively measured in the patients' plasma. Results of the drug concentration distribution of TKIs in NSCLC patients are shown in Figure 3.

The drug exposure in vivo has a close relation with the treatment efficacy and/or adverse reaction, and it was still the key point for the clinical optimization of drug dose [17–21]. There were differences in drug absorption, distribution, metabolism, and excretion between patients and within patients, and several studies in recent years were also reported [22–24]. This makes monitoring the concentration of TKI drugs' exposure in vivo particularly significant, and the method is an appropriate effective detection technology to increase the treatment efficacy and/or adverse reaction in the process of individualized dosing administration. Due to the limitation of objective conditions, the number of enrolled patients was small, due to which the relationship between the exposure and treatment efficacy could not be determined.

## 4. Conclusion

A simple, rapid, and sensitive method for the simultaneous determination of TKIs (including gefitinib, erlotinib, crizotinib, afatinib, osimertinib, and icotinib) in human plasma from NSCLC patients by the HPLC-MS/MS method was developed and validated. The analytical time was 7 min for six analytes after optimizing detection conditions, and the sample pretreatment method was simple, rapid, and economical. This method was suitable for clinical therapeutic drug monitoring to obtain a better treatment outcome.

## Figures and Tables

**Table 1:** Optimized mass spectrometry parameters of six analytes and IS.

Analytes	Ionization mode (±)	Precursor ions (m/z)	Fragmentor (V)	Collision energy (V)	Product ions (m/z)
Gefitinib	+	447.2	90	22	128.2
Icotinib	+	392.1	155	32	304.2
Afatinib	+	486.2	140	32	371.1
Erlotinib	+	394.2	100	30	278.1
Crizotinib	+	450.2	140	26	260.1
Osimertinib	+	499.9	105	26	72.0
Carbamazepine (IS)	+	237.1	115	19	194.1

**Table 2:** Linearity regression parameters of six analytes.

Analytes	Regression type	Linear range	Weighing factor	Regression equations	*R*
Gefitinib	Linearity	10.0–1000.0	1/*χ*^2^	*y* = 0.0025 ∗ *x* − 0.0029	0.9915
Icotinib	Linearity	50.0–4000.0	1/*χ*^2^	*y* = 0.0011 ∗ *x* − 0.0129	0.9981
Erlotinib	Linearity	50.0–4000.0	1/*χ*^2^	*y* = 0.0024 ∗ *x* − 0.0275	0.9986
Crizotinib	Linearity	10.0–1000.0	1/*χ*^2^	*y* = 0.0193 ∗ *x* − 0.4494	0.9940
Osimertinib	Linearity	10.0–1000.0	1/*χ*^2^	*y* = 0.0016 ∗ *x* − 0.0023	0.9911
Afatinib	Linearity	5.0–400.0	1/*χ*^2^	*y* = 0.0141 ∗ *x* − 0.4145	0.9934

**Table 3:** Precision and accuracy of the LLOQ of six analytes' calibration curves.

Analytes	Nominal concentration (ng/mL)	Measured concentration (ng/ml) ± SD	Precision RSD (%)	Accuracy mean (%)
Gefitinib	10	8.82 ± 0.29	6.31	96.22
Icotinib	50	50.22 ± 0.91	1.81	100.44
Erlotinib	50	49.01 ± 0.92	1.87	98.01
Crizotinib	10	10.07 ± 0.36	7.27	101.39
Osimertinib	10	9.75 ± 0.21	4.31	95.15
Afatinib	5	5.30 ± 0.31	5.76	105.98

**Table 4:** Extraction recovery and matrix effect of six analytes (%) (*n* = 3).

Analytes	Nominal concentration (ng/mL)	Extraction recovery		Matrix effect	
		Mean (%) ± SD	RSD (%)	Mean (%) ± SD	RSD (%)
Gefitinib	20	91.18 ± 3.49	3.08	114.09 ± 7.4	2.56
	100	80.75 ± 3.34		113.83 ± 8.88	
	500	97.18 ± 1.13		108.98 ± 11.45	

Icotinib	100	81.41 ± 1.33	3.11	107.58 ± 4.28	3.49
	500	93.89 ± 3.91		100.91 ± 2.91	
	2000	96.02 ± 1.62		101.85 ± 5.85	

Erlotinib	100	85.24 ± 3.33	3.00	108.67 ± 8.22	5.49
	500	90.10 ± 1.8		101.26 ± 2.47	
	2000	85.85 ± 1.99		97.63 ± 5.90	

Crizotinib	20	76.66 ± 6.33	2.71	109.91 ± 12.21	5.31
	100	77.75 ± 5.15		100.98 ± 7.88	
	500	82.10 ± 3.87		96.83 ± 11.76	

Osimertinib	20	82.25 ± 7.63	2.92	100.58 ± 4.94	2.97
	100	90.53 ± 2.48		102.72 ± 5.27	
	500	81.76 ± 4.03		96.85 ± 10.60	

Afatinib	10	85.20 ± 1.38	3.12	102.56 ± 17.39	2.91
	50	90.55 ± 8.06		108.45 ± 15.34	
	200	95.69 ± 8.61		107.08 ± 9.03	

RSD was calculated using the IS-normalized matrix and recovery factors.

**Table 5:** Inter- and intra-accuracy and precision of six analytes (*n* = 5).

Analytes	Nominal concentration (ng/ml)	Interday			Intraday		
		Measured concentration (mean ± SD)	Accuracy (RE %)	Precision (RSD %)	Measured concentration (mean ± SD)	Accuracy (RE %)	Precision (RSD %)
Gefitinib	20	17.70 ± 0.66	−11.5	3.75	17.32 ± 0.26	−13.4	1.51
	100	87.81 ± 1.81	−12.19	2.06	87.77 ± 2.22	−12.23	2.53
	500	552.89 ± 15.82	10.59	2.86	565.21 ± 9.26	13.04	1.63

Icotinib	100	91.09 ± 0.93	−8.91	1.02	91.56 ± 0.49	−8.44	0.53
	500	439.77 ± 12.18	−12.05	2.77	426.70 ± 1.82	−14.66	0.42
	2000	1919.82 ± 39.30	−0.40	2.04	1966.42 ± 27.49	−1.68	1.39

Erlotinib	100	87.59 ± 1.14	−12.41	1.31	88.94 ± 0.82	−11.06	0.93
	500	441.47 ± 6.58	−11.71	1.50	450.70 ± 1.51	−9.86	0.33
	2000	1887.25 ± 94.36	−5.64	2.19	1933.51 ± 37.33	−3.25	1.93

Crizotinib	20	18.17 ± 0.62	−9.15	3.42	18.55 ± 0.92	−7.25	4.97
	100	91.67 ± 3.11	−8.33	3.39	88.67 ± 1.71	−11.33	1.92
	500	538.54 ± 12.68	7.71	2.35	538.57 ± 4.57	7.71	0.85

Osimertinib	20	18.72 ± 0.92	−6.40	4.92	18.52 ± 0.46	−7.40	2.53
	100	85.36 ± 2.96	−14.64	3.47	85.24 ± 4.95	−14.76	5.81
	500	556.91 ± 11.80	11.38	2.12	561.76 ± 15.24	12.35	2.71

Afatinib	10	9.92 ± 0.53	−0.80	5.41	9.76 ± 0.19	−2.40	1.94
	50	53.78 ± 2.28	7.56	4.24	51.54 ± 1.74	3.08	3.37
	200	217.83 ± 4.73	8.92	2.17	220.65 ± 4.71	10.33	2.13

**Table 6:** Results of stability of six analytes (*n* = 3).

Analytes	Nominal concentration (ng/mL)	Freeze-thaw stability		12 h at room temperature		24 h in the autosampler		Long-term stability (1 month)	
		Measured concentration (mean)	Accuracy (RE %)	Measured concentration (mean)	Accuracy (RE %)	Measured concentration (mean)	Accuracy (RE %)	Measured concentration (mean)	Accuracy (RE %)
Gefitinib	20	17.58	−12.10	17.46	−12.70	17.56	−12.20	17.47	−12.65
	100	88.85	−11.15	90.03	−9.97	86.11	13.89	88.34	−11.66
	500	543.30	8.66	553.20	10.64	556.10	11.22	541.33	8.27

Icotinib	100	91.87	−8.13	90.71	−9.29	91.04	−8.96	89.09	−10.91
	500	447.00	−10.60	446.90	−10.62	441.40	−11.72	433.56	−13.28
	2000	1920.00	−4.00	1972.00	−1.40	1969.00	−1.55	1950.43	−2.47

Erlotinib	100	87.75	−12.25	89.76	−10.24	87.53	−12.47	86.34	−13.66
	500	440.90	−11.82	450.70	−9.86	440.90	−11.82	430.55	−13.89
	2000	1887.00	−5.65	1950.01	−2.50	1938.00	−3.10	1840.11	−7.99

Crizotinib	20	18.05	−9.75	18.92	−5.40	18.94	−5.30	17.32	−13.40
	100	91.86	−8.14	93.22	−6.78	94.22	−5.78	93.21	−6.79
	500	541.90	8.38	550.80	10.16	550.70	10.14	544.13	8.83

Osimertinib	20	19.01	−4.95	17.80	−11.00	17.55	−12.50	19.02	−4.90
	100	88.06	−11.94	87.57	−12.43	85.95	−14.05	87.23	−12.77
	500	551.30	10.26	561.70	12.34	549.40	9.88	561.07	12.21

Afatinib	10	10.10	9.10	10.71	7.10	10.07	0.70	10.21	2.1
	50	53.57	7.14	53.56	7.12	51.78	3.56	49.03	−1.94
	200	219.92	9.96	219.97	9.85	218.24	9.12	209.23	4.627

## Data Availability

All data are included within the article and the supplementary materials.

## Supplementary Materials

Supplementary Materials To test the applicability of this method, 25 plasma samples were collected from 25 NSCLC patients who were treated with one of the six TKIs. Icotinib (n = 9), osimertinib (n = 7), gefitinib (n = 3), crizotinib (n = 3), afatinib (n = 2), and erlotinib (n = 1) were collected from clinical therapeutics patients. The data results of drug valley concentration values in human plasma of six TKIs are shown as supplementary materials. Table 1: plasma concentration of TKIs was determined by oral administration.
