# Supplementary material for: Simultaneous and Rapid Determination of Six Tyrosine Kinase Inhibitors in Patients with Non-Small Cell Lung Cancer Using HPLC-MS/MS
Source: Int J Anal Chem. 2021 Sep 17;2021:5524361. doi: 10.1155/2021/5524361 (PMC8463214; doi:10.1155/2021/5524361)
Supplement: Supplementary Materials — To test the applicability of this method, 25 plasma samples were collected from 25 NSCLC patients who were treated with one of the six TKIs. Icotinib (n = 9), osimertinib (n = 7), gefitinib (n = 3), crizotinib (n = 3), afatinib (n = 2), and erlotinib (n = 1) were collected from clinical therapeutics patients. The data results of drug valley concentration values in human plasma of six TKIs are shown as supplementary materials. Table 1: plasma concentration of TKIs was determined by oral administration. [file 5524361.f1.doc]

**Supplementary Materials**

The simple, rapid and sensitive analysis method established in this paper was used for concentration determination, and the data results of drug valley concentration values in human plasma of six TKIs were shown in Table 1. ( *Supplementary Materials* ).

Table 1 Plasma concentration of TKIs was determined by oral administration:

| Number of patients | Plasma concentration  (ng/mL) | Mean concentration  (ng/ml) | Standard deviation  (±SD) |
| --- | --- | --- | --- |
| Gefitinib (n=3) | 600.07 | 655.90 | 65.39 |
|  | 639.79 |  |  |
|  | 727.84 |  |  |
| Erlotinib (n=1) | 78.43 | / | / |
| Crizotinib (n=3) | 272.80 | 274.08 | 7.00 |
|  | 267.80 |  |  |
|  | 281.63 |  |  |
| Afatinib (n=2) | 30.50 | 31.42 | 1.30 |
|  | 32.40 |  |  |
| Osimertinib (n=7) | 272.22 | 218.72 | 78.39 |
|  | 282.06 |  |  |
|  | 272.74 |  |  |
|  | 276.59 |  |  |
|  | 173.66 |  |  |
|  | 175.99 |  |  |
|  | 77.75 |  |  |
| Icotinib (n=9) | 724.48 | 1366.72 | 908.67 |
|  | 737.55 |  |  |
|  | 2504.75 |  |  |
|  | 2532.16 |  |  |
|  | 2100.87 |  |  |
|  | 2094.43 |  |  |
|  | 519.14 |  |  |
|  | 515.03 |  |  |
|  | 572.1 |  |  |
